# Supplementary figures and images for: XAB2 dynamics during DNA damage-dependent transcription inhibition
Source: eLife. 2022 Jul 26;11:e77094. doi: 10.7554/eLife.77094 (PMC9436415; doi:10.7554/eLife.77094)

Figure 1B

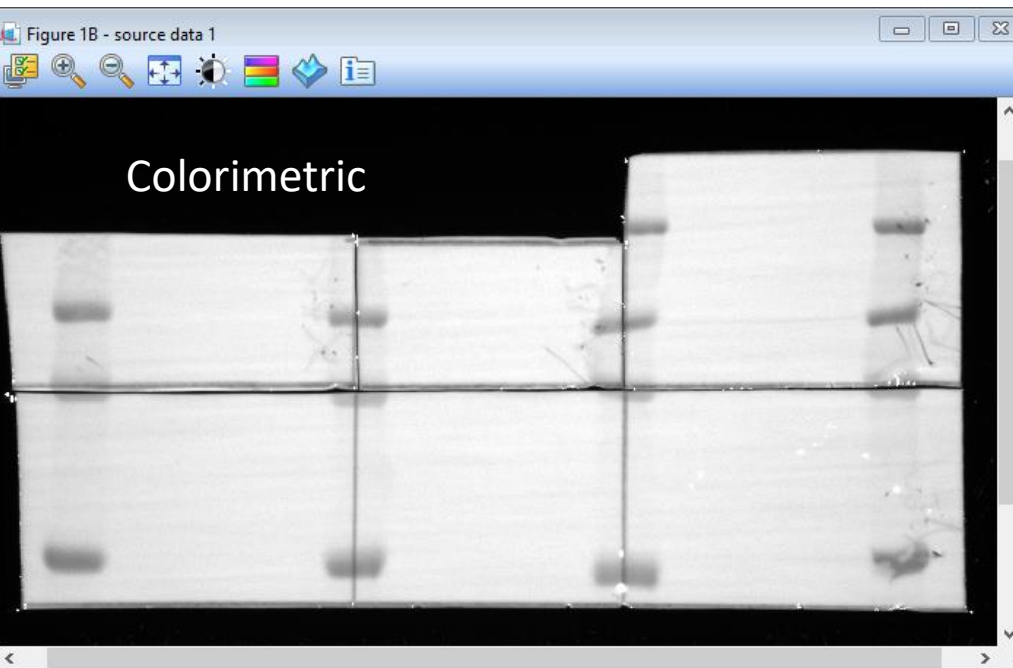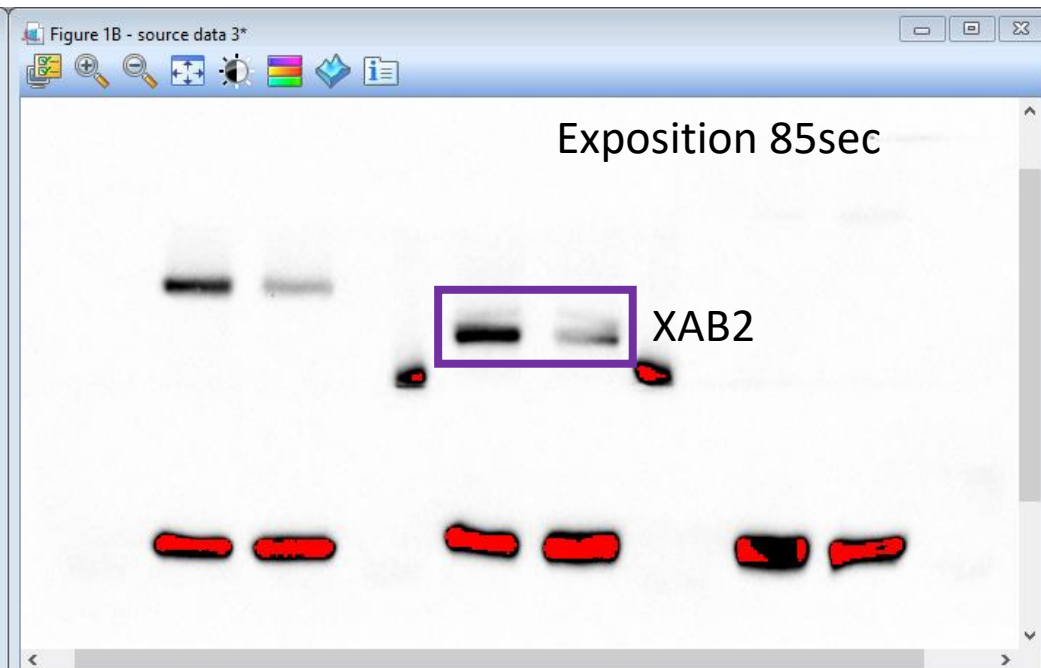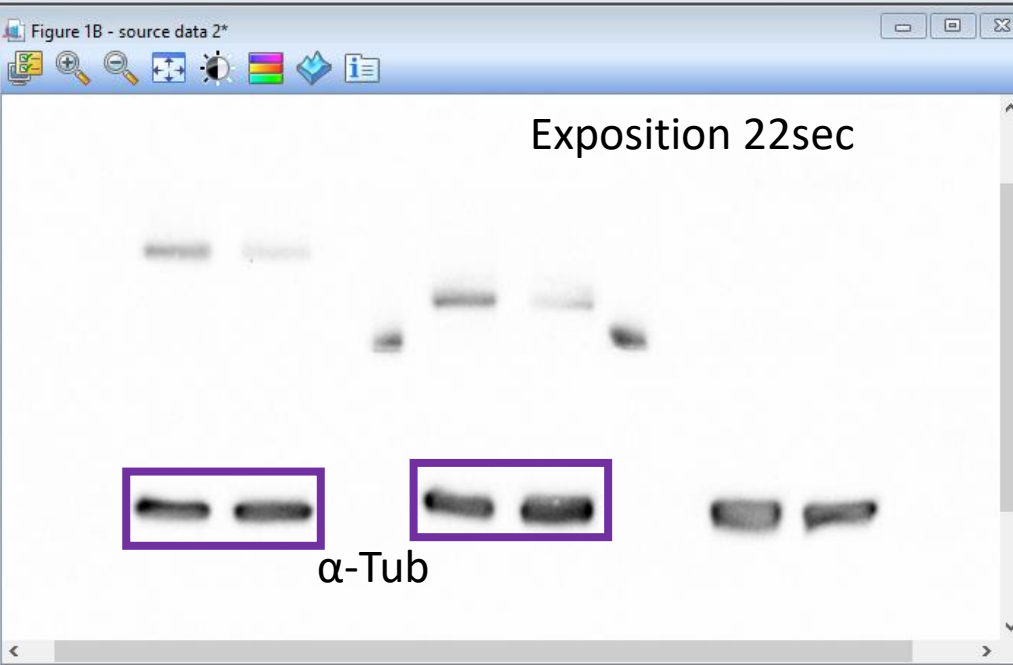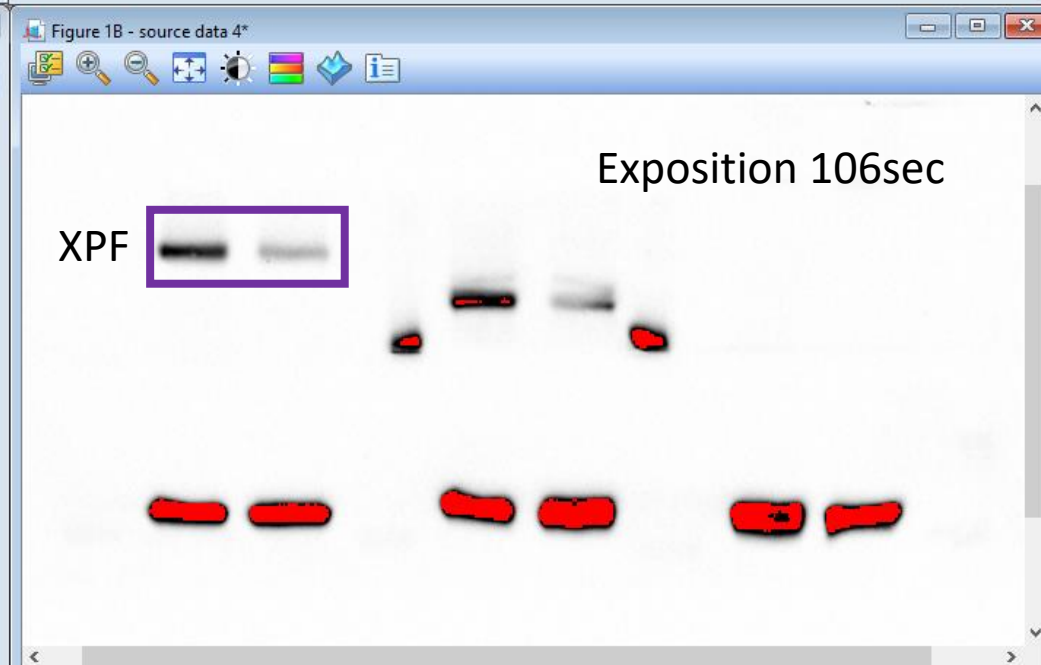

Supplement: Figure 1—source data 4. [file elife-77094-fig1-data4.pdf]

**Figure 5B**

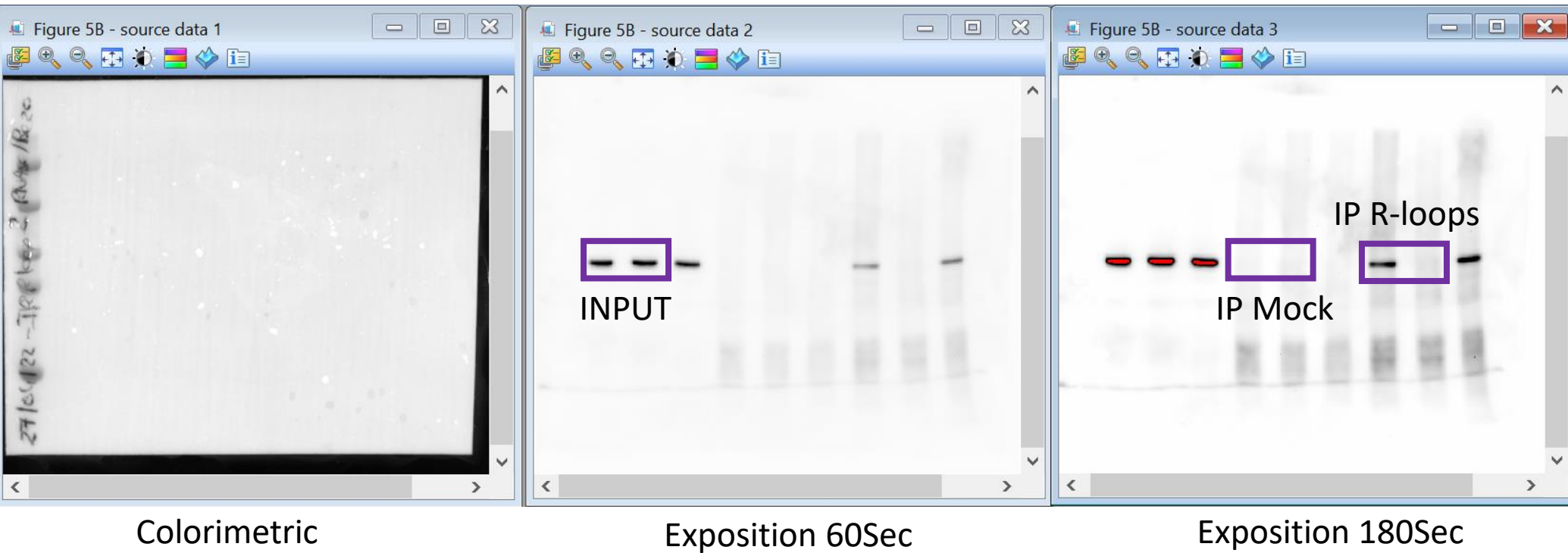

Supplement: Figure 5—source data 5. [file elife-77094-fig5-data5.pdf]
