# Supplementary material for: XAB2 dynamics during DNA damage-dependent transcription inhibition
Source: eLife. 2022 Jul 26;11:e77094. doi: 10.7554/eLife.77094 (PMC9436415; doi:10.7554/eLife.77094)

Figure 2 – figure supplement 2C

Colorimetric 1

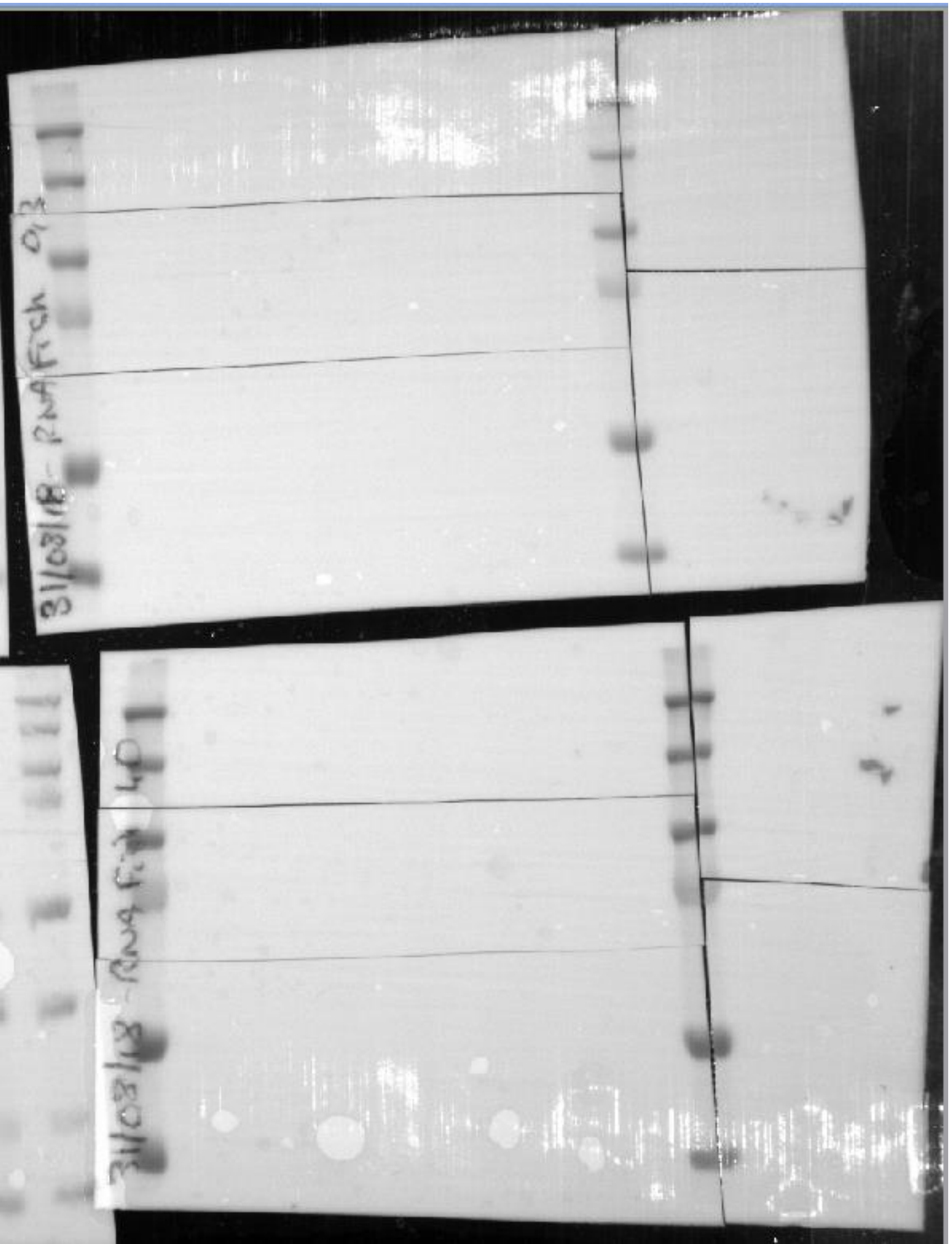

Exposition 30sec

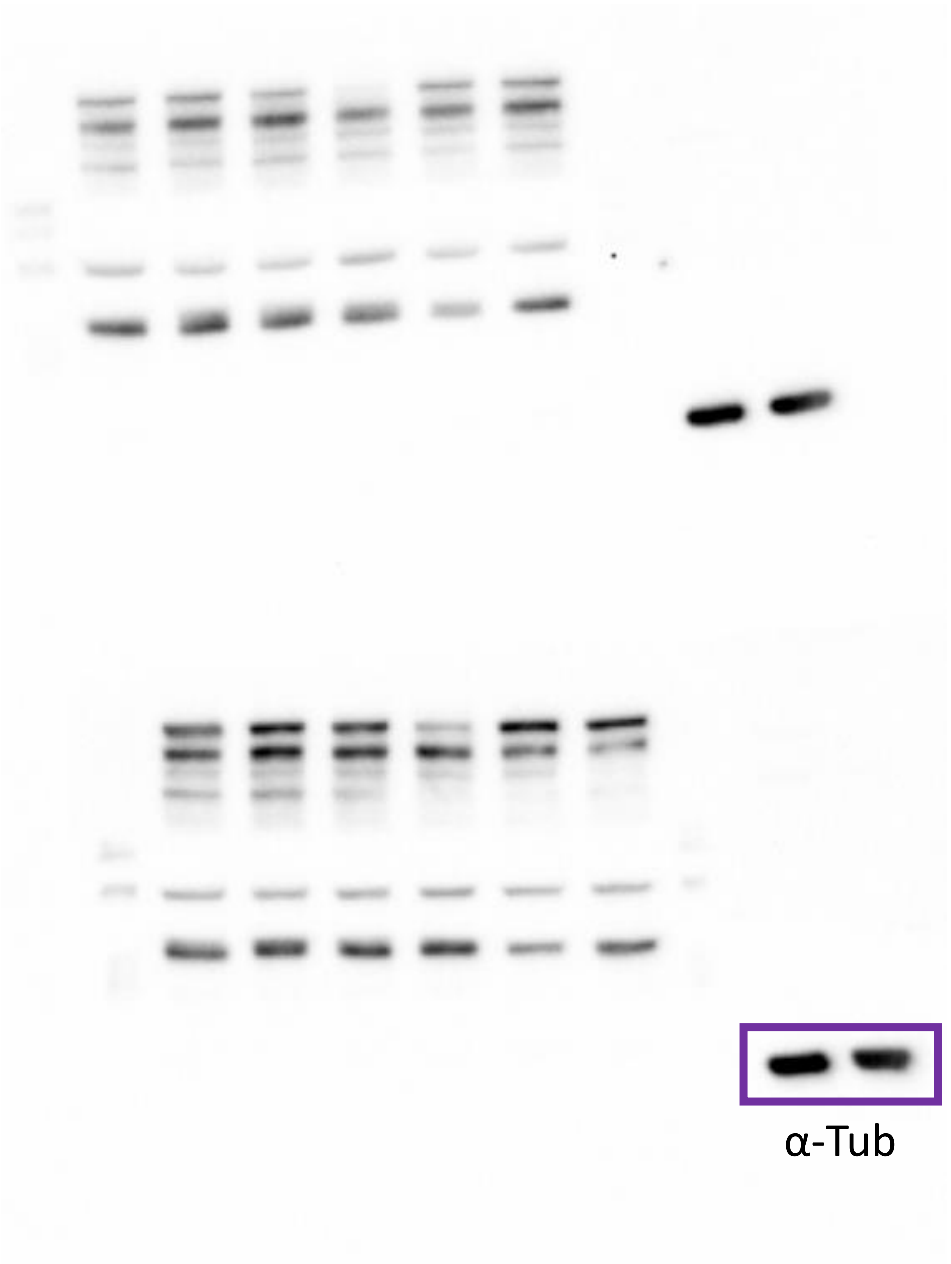

Figure 2 – figure supplement 2C

Colorimetric 2

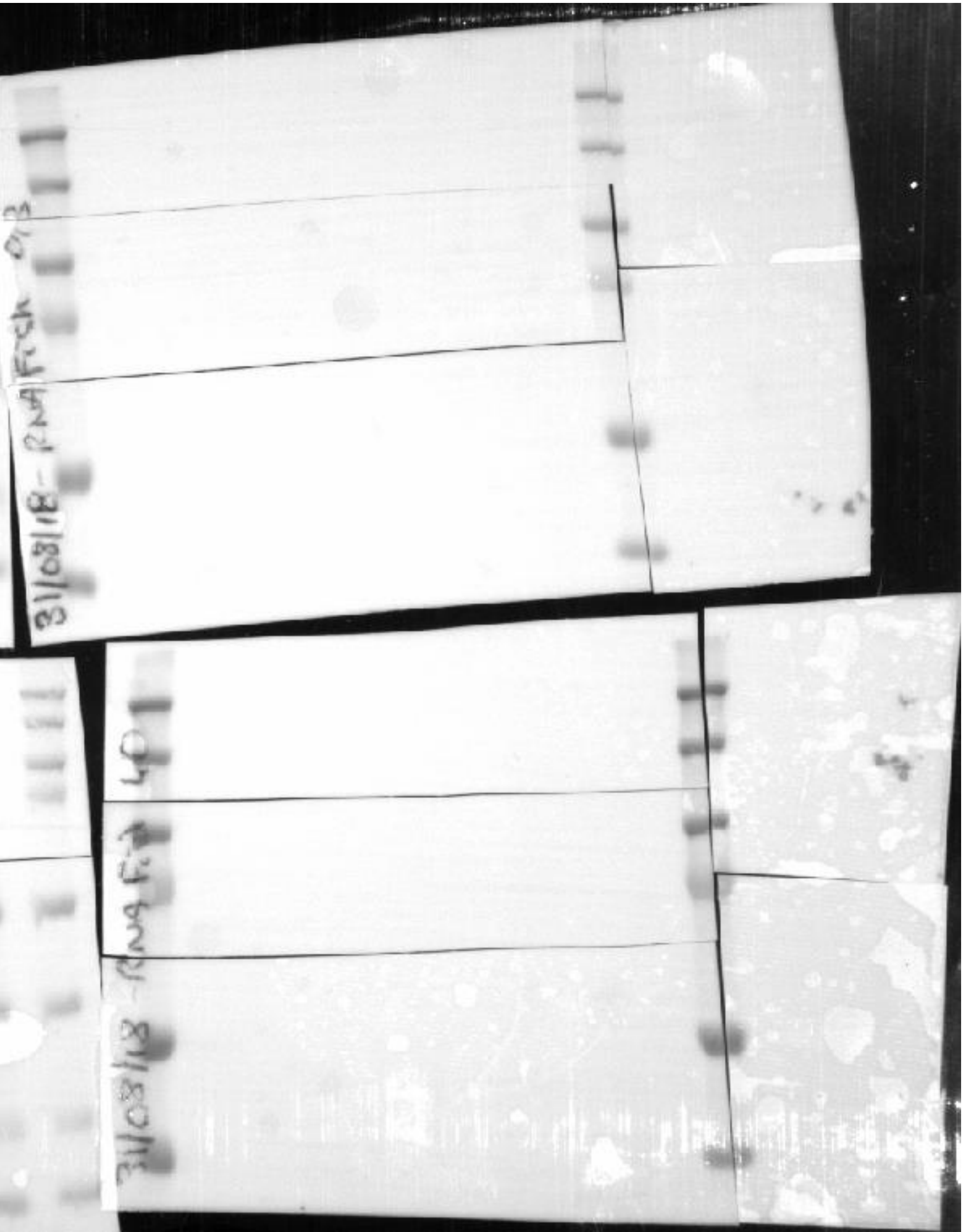

Exposition 140sec – ECL++

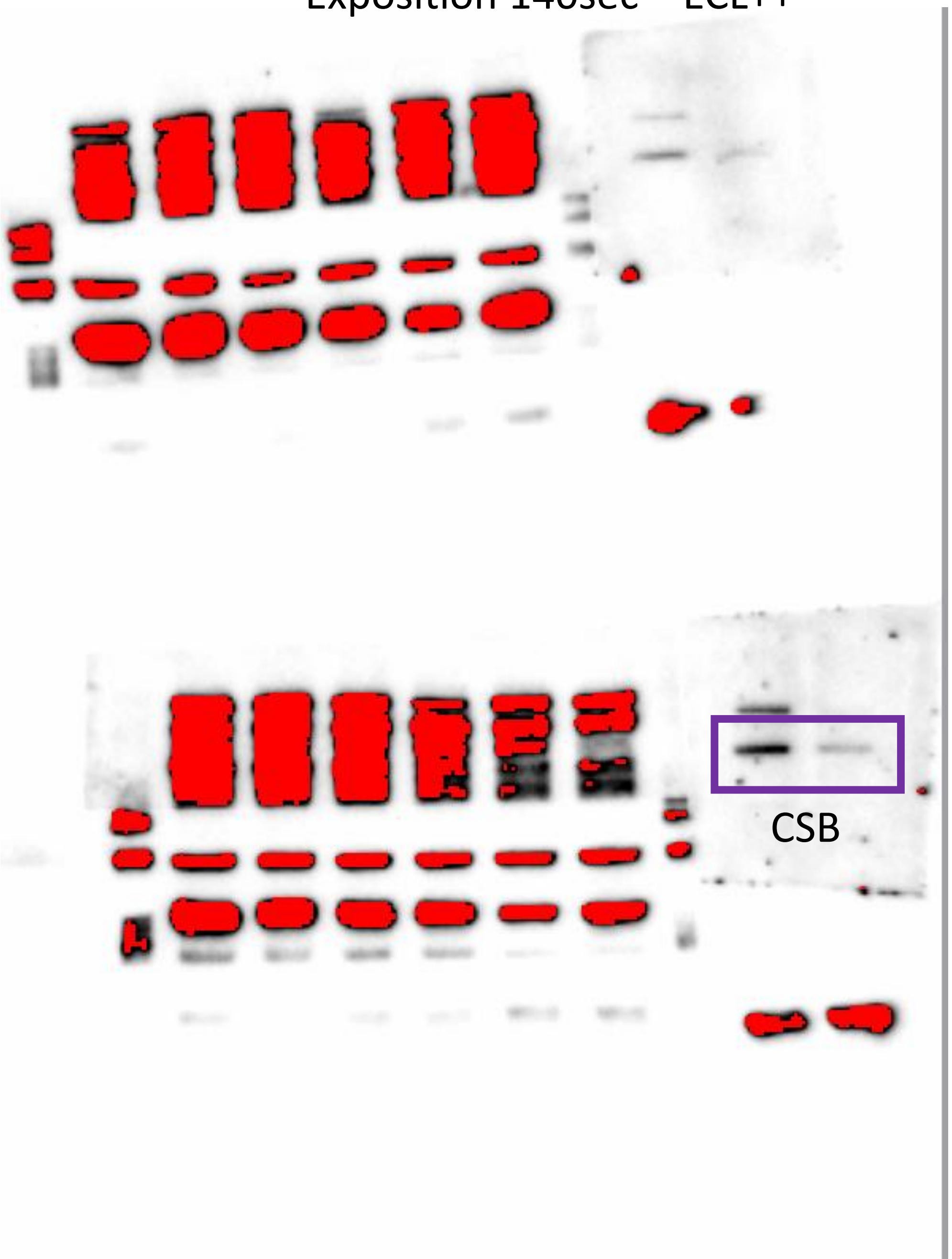

Supplement: Figure 2—figure supplement 2—source data 2. [file elife-77094-fig2-figsupp2-data2.pdf]
