# Supplementary material for: XAB2 dynamics during DNA damage-dependent transcription inhibition
Source: eLife. 2022 Jul 26;11:e77094. doi: 10.7554/eLife.77094 (PMC9436415; doi:10.7554/eLife.77094)

Figure 3 – figure supplement 1C

Colorimetric 1

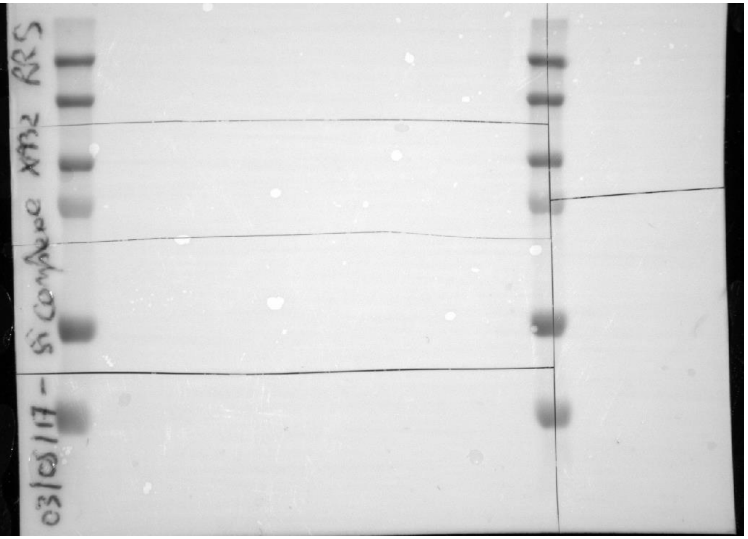

Exposition 60sec

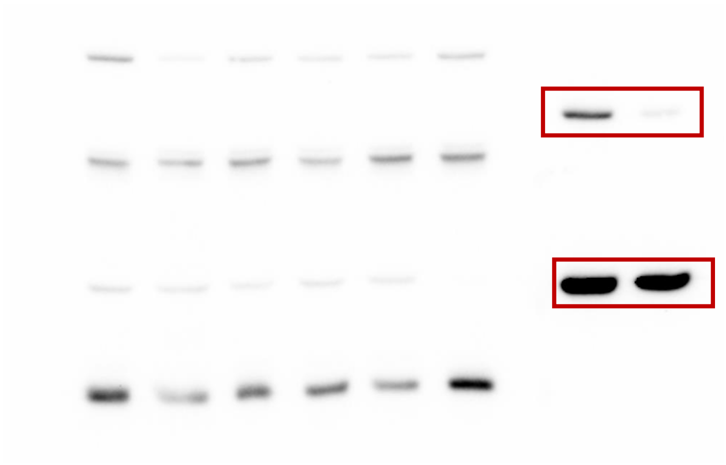

XPF

α-tub

Figure 3 – figure supplement 1C

Colorimetric 1

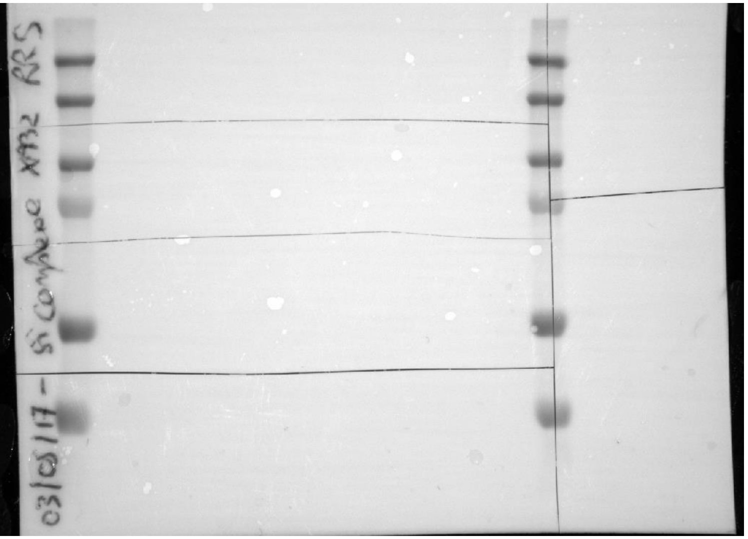

Exposition 60sec

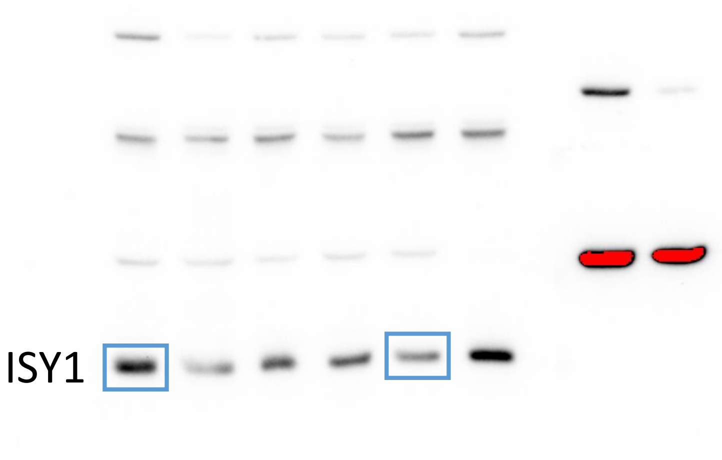

Exposition 120sec

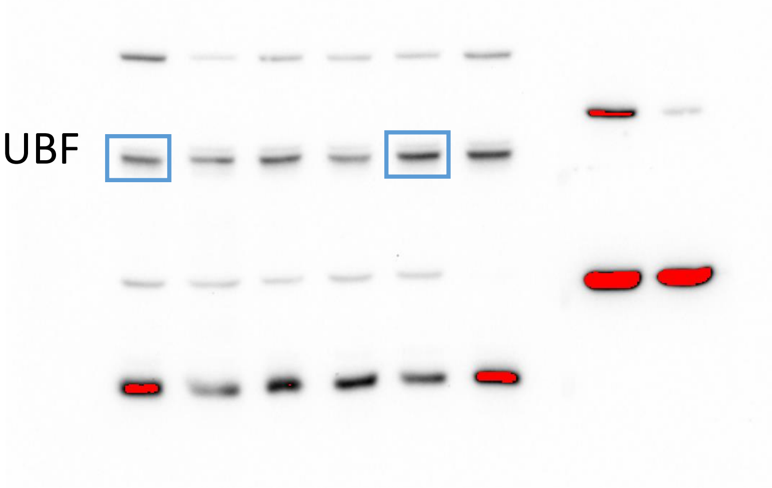

Figure 3 – figure supplement 1C

Colorimetric 1

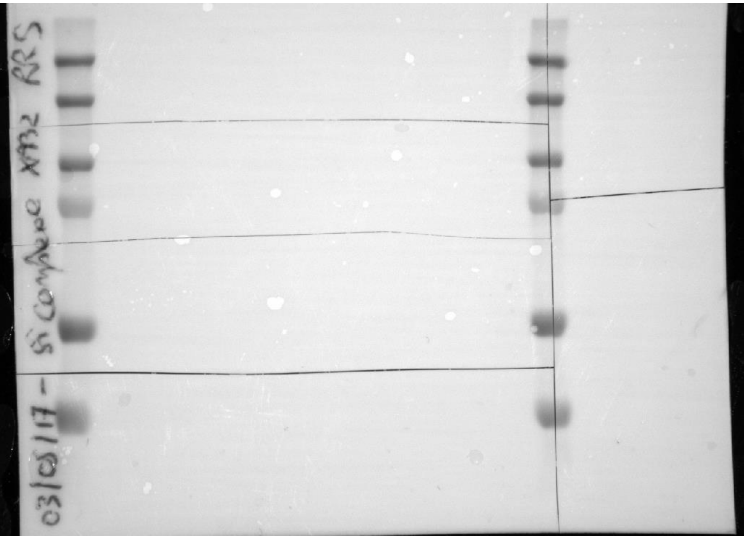

Exposition 180sec

AQR

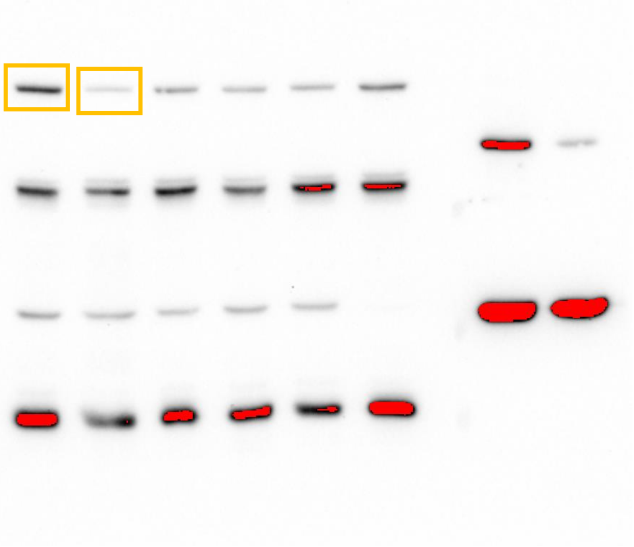

Exposition 120sec

UBF

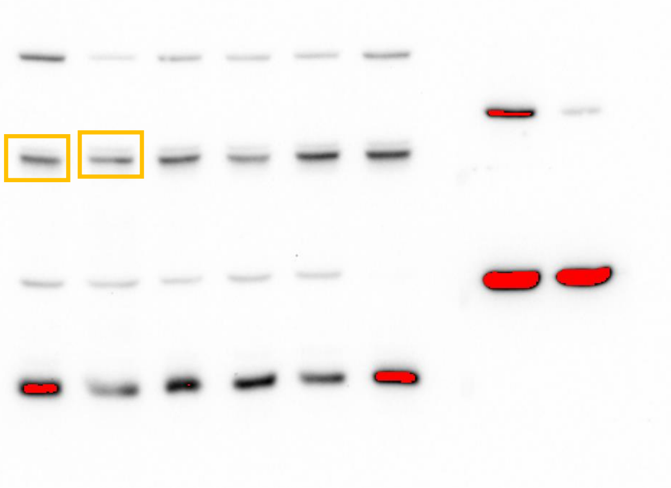

Figure 3 – figure supplement 1C

Colorimetric 1

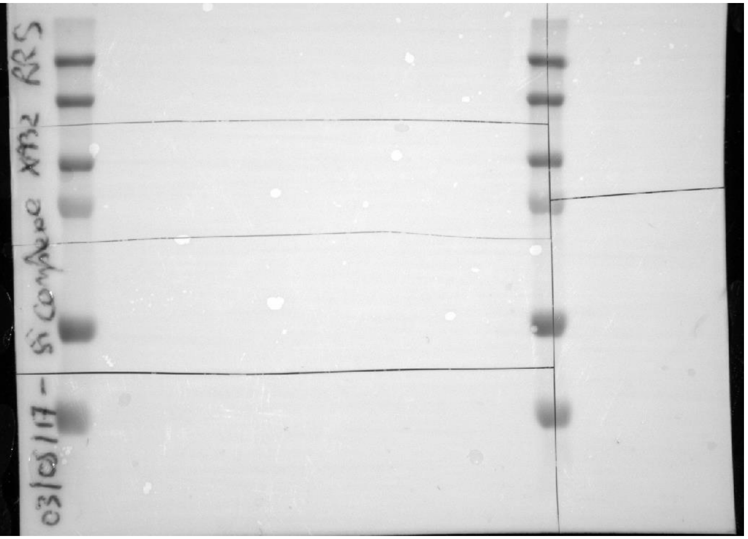

Exposition 600sec

PRP19

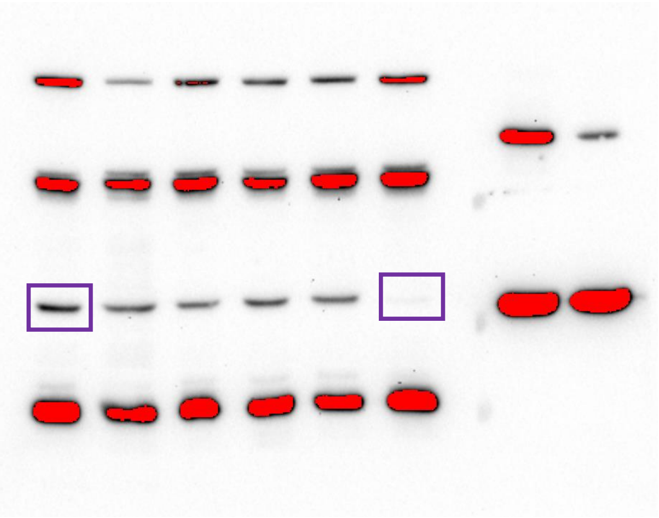

Exposition 120sec

UBF

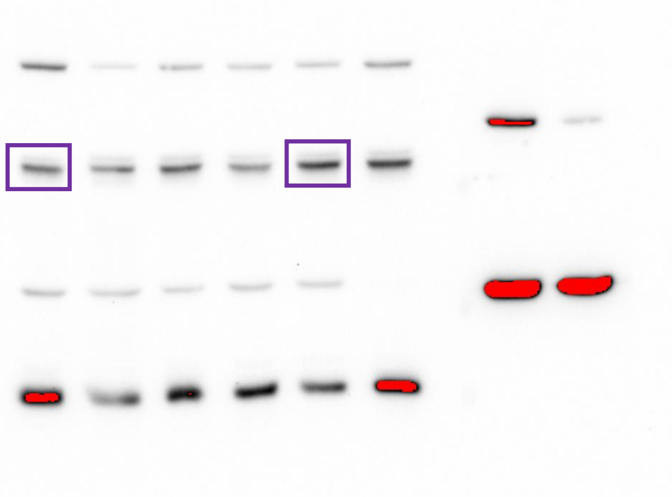

Figure 3 – figure supplement 1C

Colorimetric 2

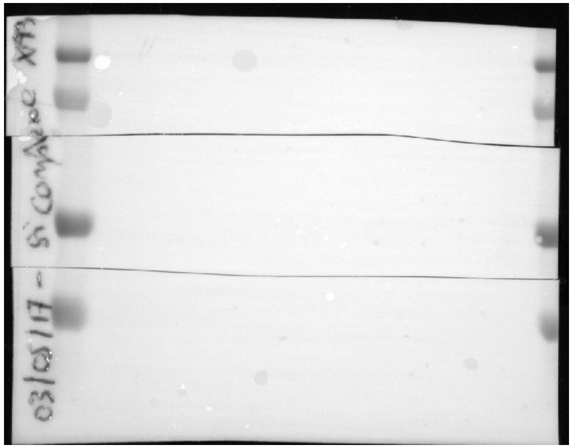

Exposition 1200sec-reblot

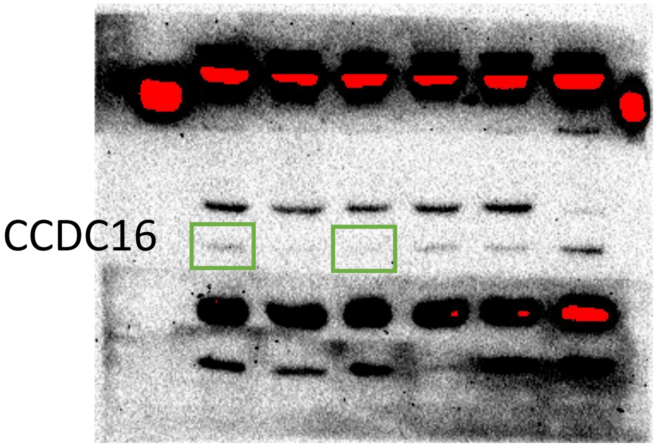

source data 7

Colorimetric 1

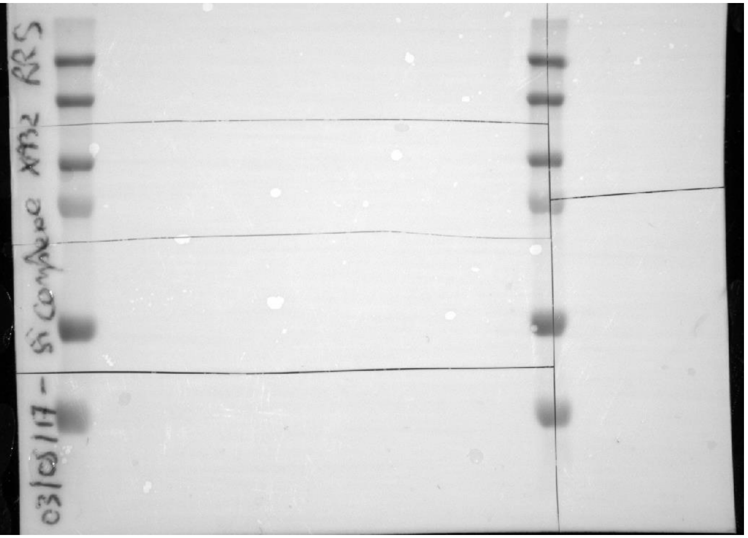

Exposition 120sec

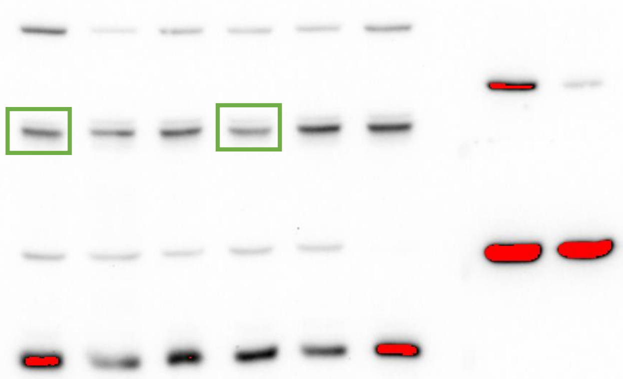

Figure 3 – figure supplement 1C

Colorimetric 2

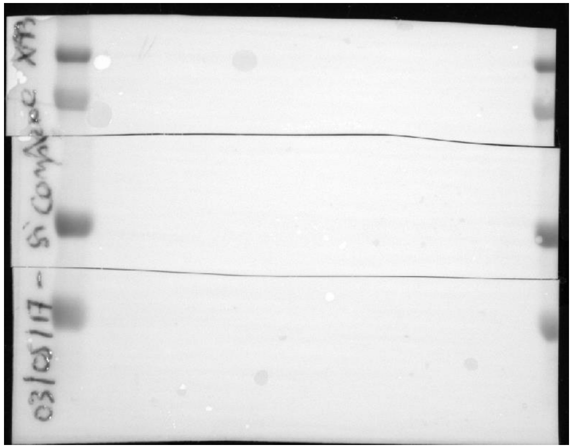

Exposition 1200sec-reblot

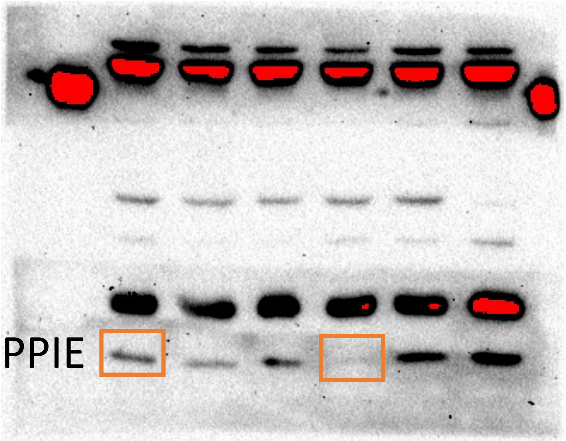

Colorimetric 1

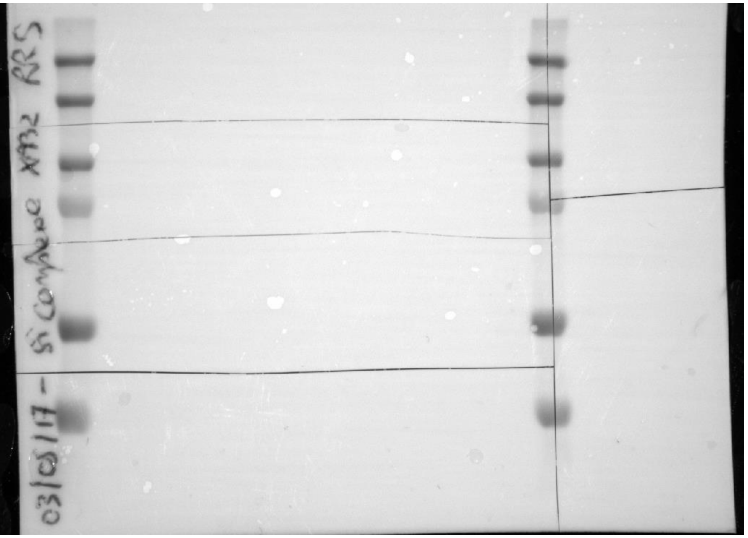

Exposition 120sec

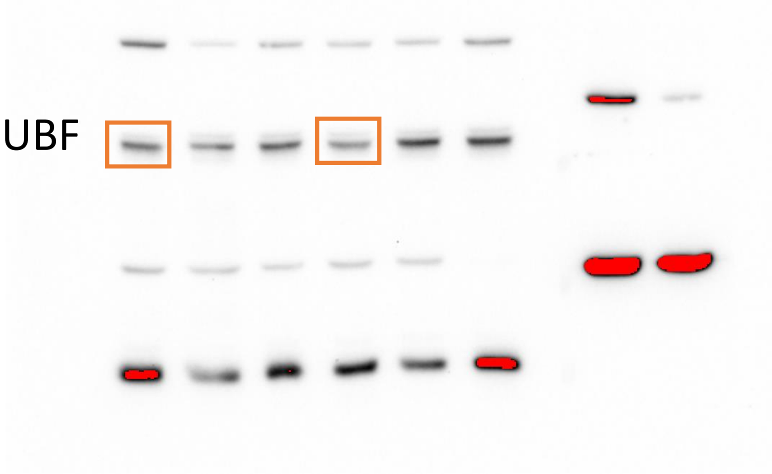

Supplement: Figure 3—figure supplement 1—source data 2. [file elife-77094-fig3-figsupp1-data2.pdf]
