# Supplementary material for: XAB2 dynamics during DNA damage-dependent transcription inhibition
Source: eLife. 2022 Jul 26;11:e77094. doi: 10.7554/eLife.77094 (PMC9436415; doi:10.7554/eLife.77094)

Figure 5A And Figure 5 – figure supplement 1A

Colorimetric

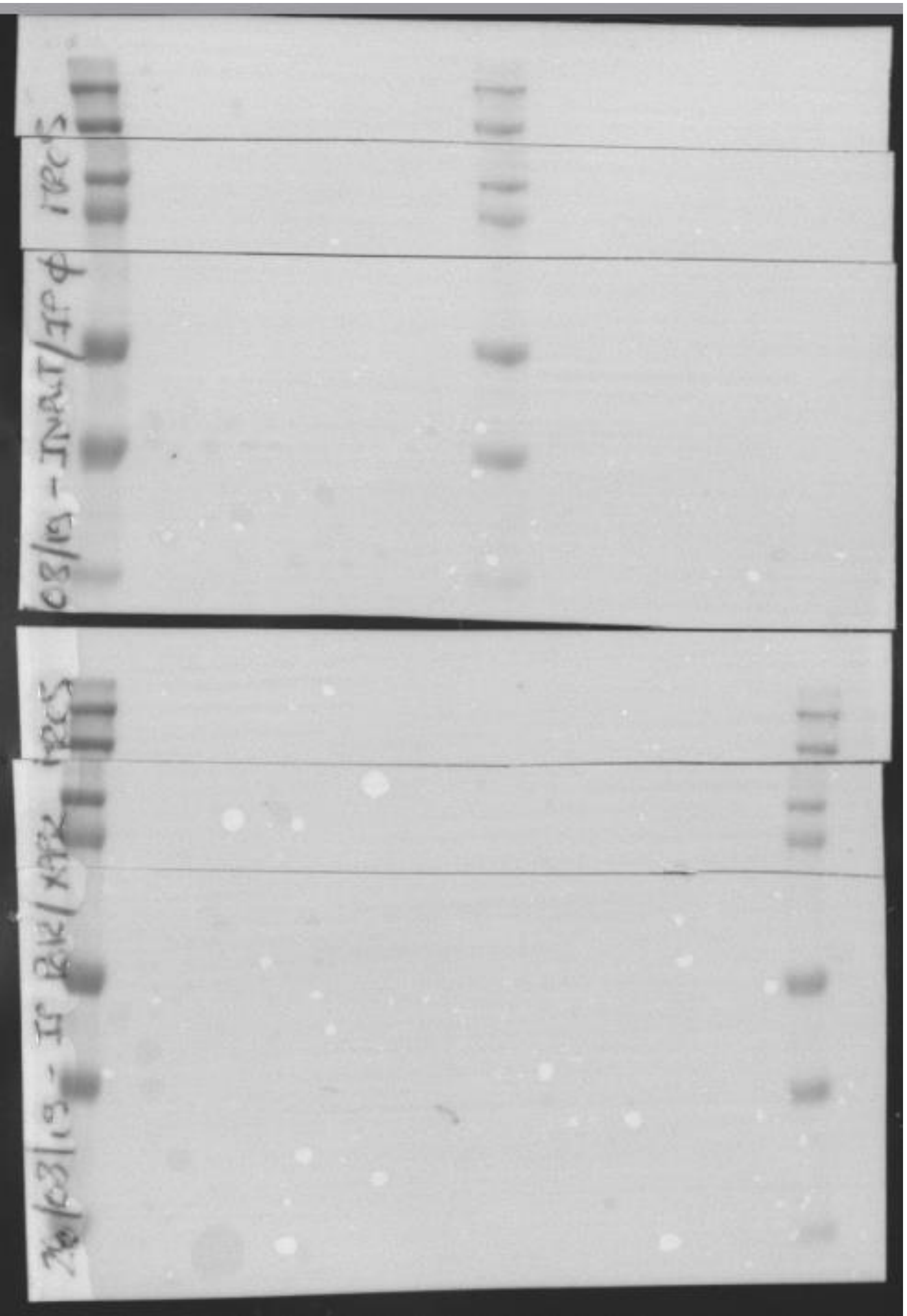

MRC5

Exposition 60sec

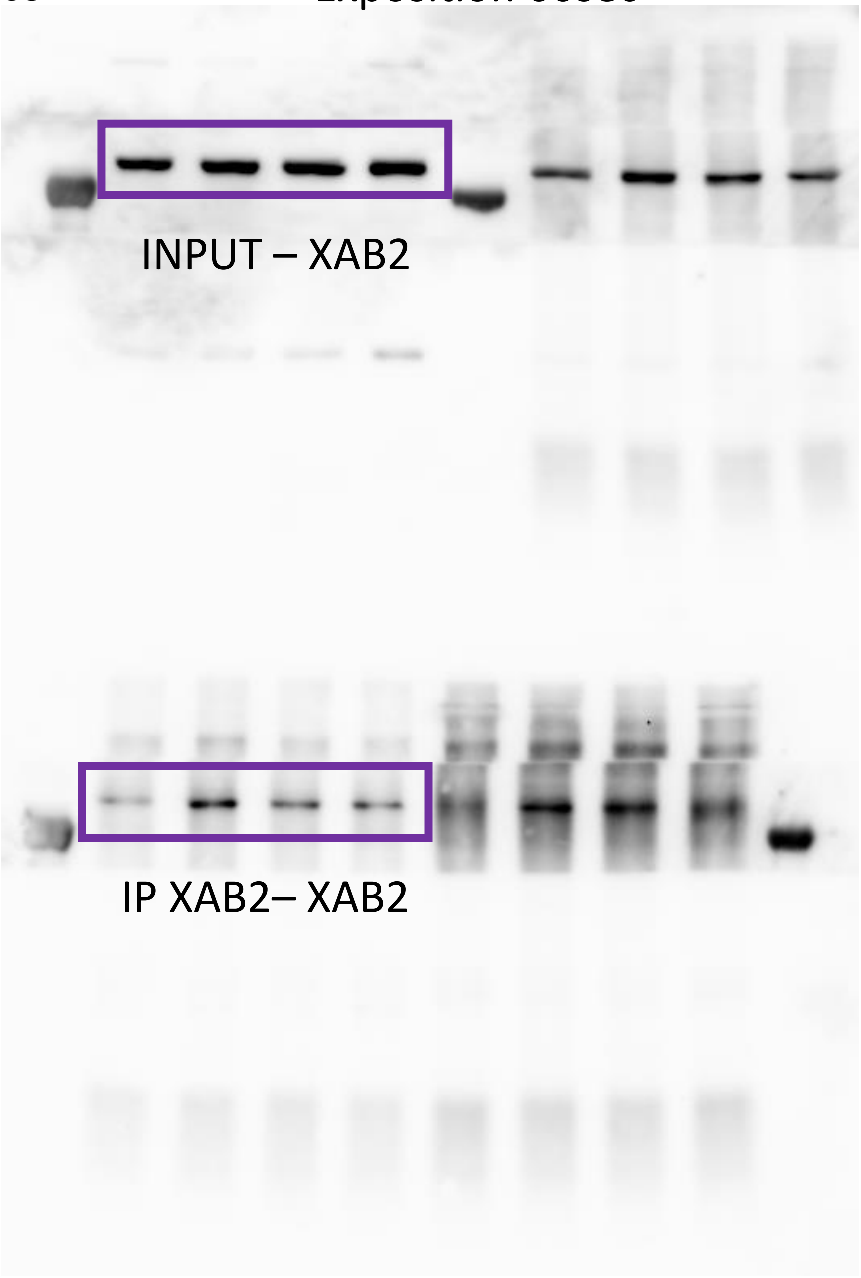

Figure 5A
CSA-/-

Colorimetric

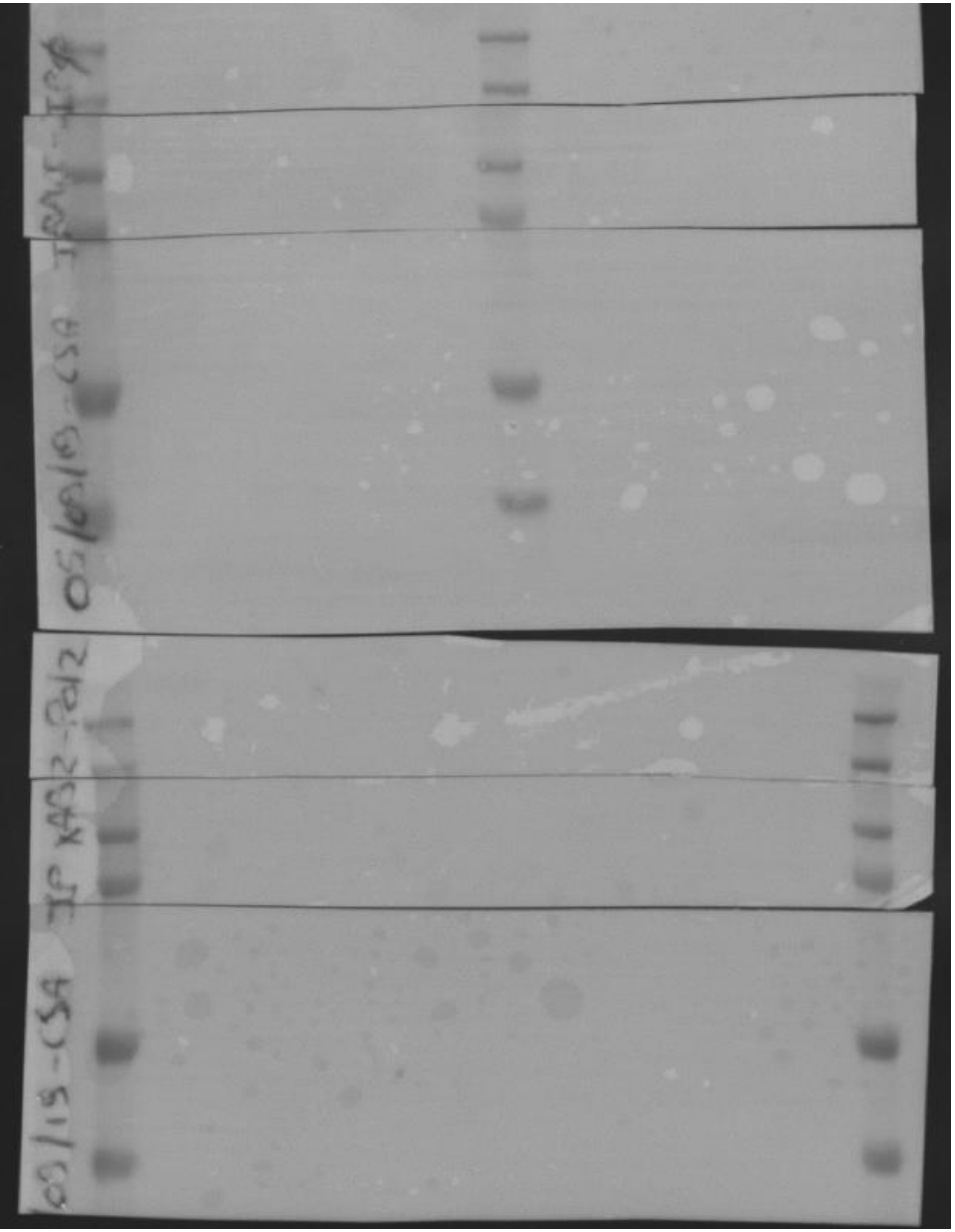

Exposition 60sec

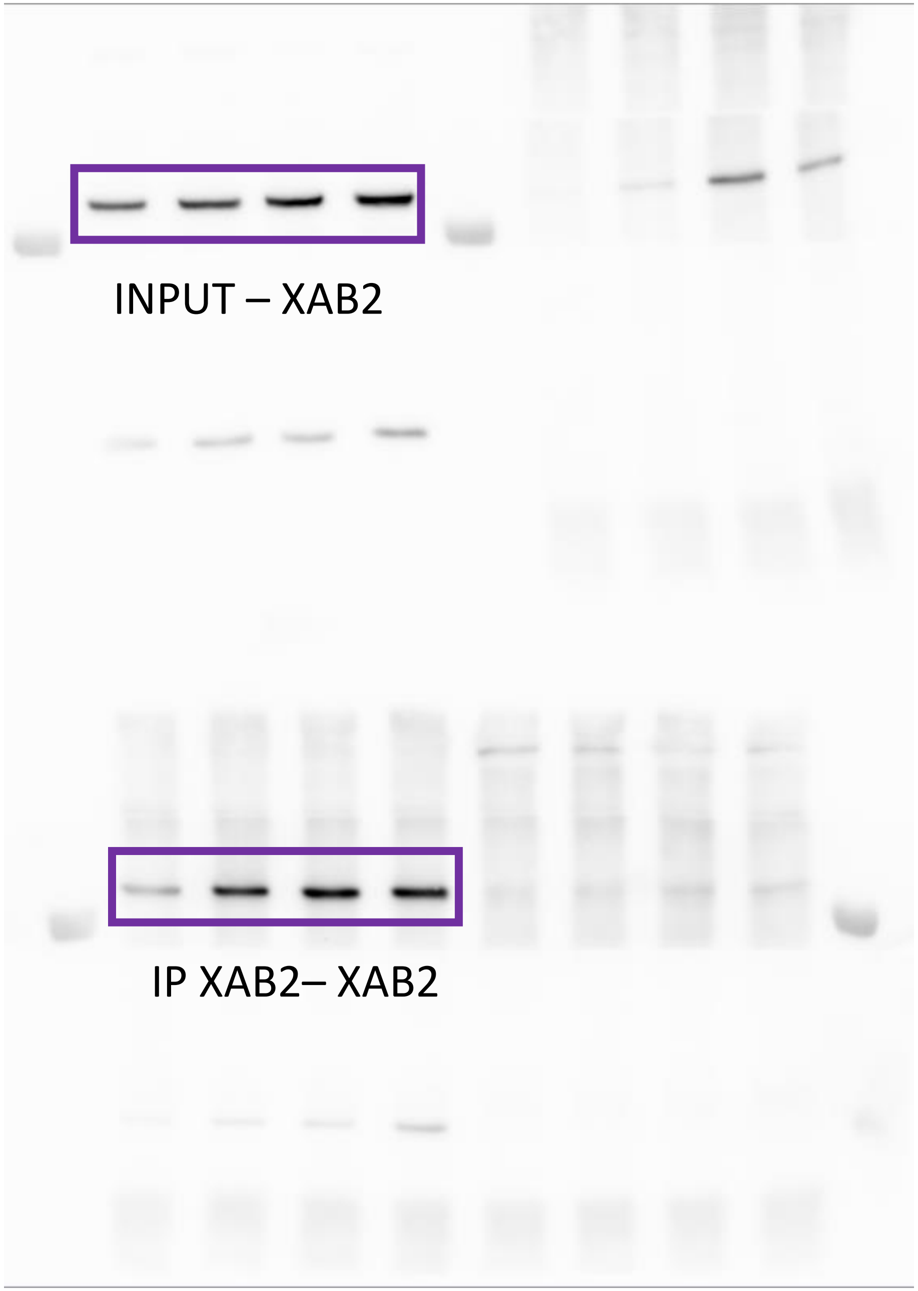

Figure 5A
CSB-/-

Colorimetric

Exposition 60sec

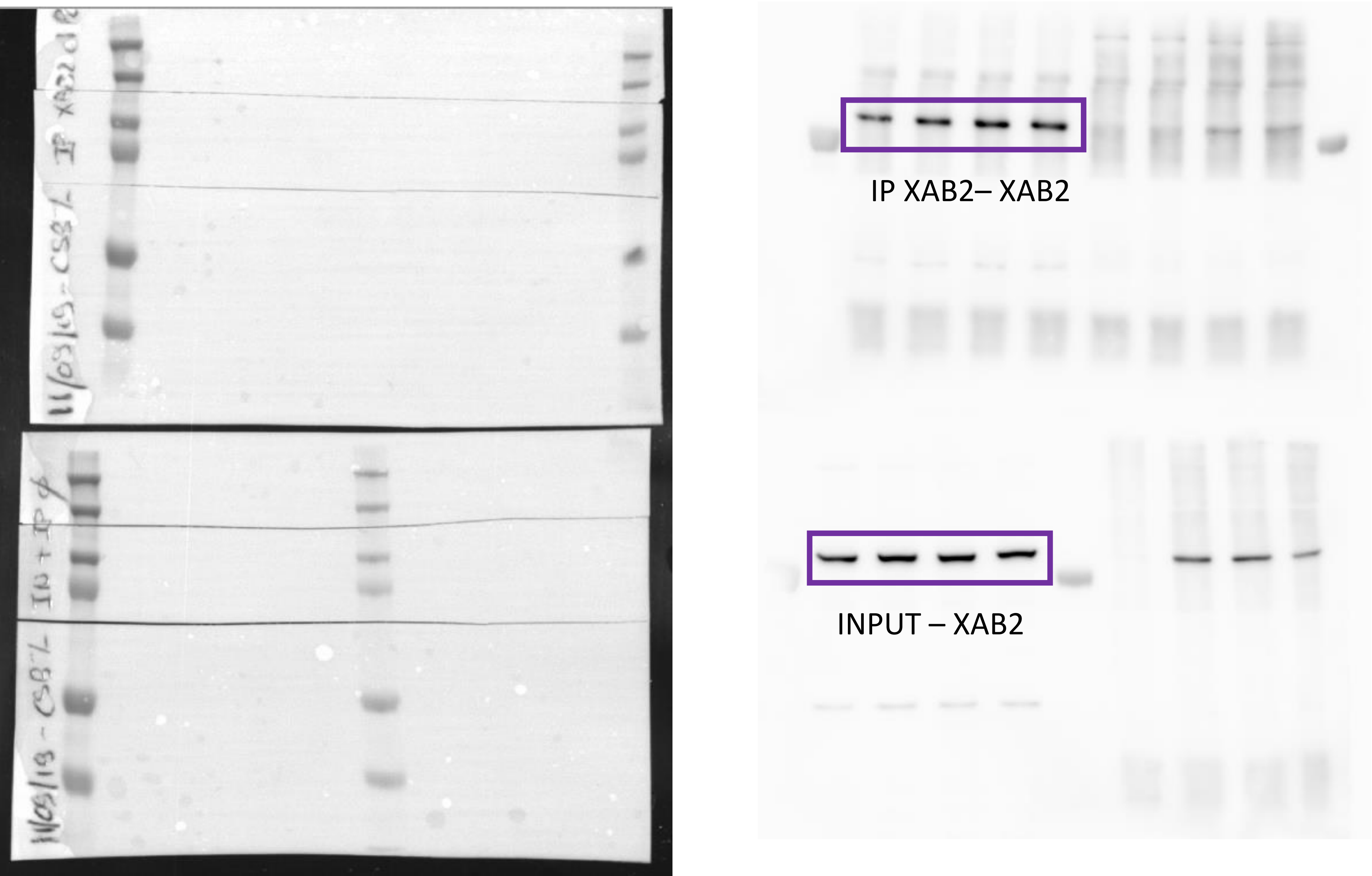

Supplement: Figure 5—source data 3. [file elife-77094-fig5-data3.pdf]
