# Supplementary material for: XAB2 dynamics during DNA damage-dependent transcription inhibition
Source: eLife. 2022 Jul 26;11:e77094. doi: 10.7554/eLife.77094 (PMC9436415; doi:10.7554/eLife.77094)

Figure 5A And Figure 5 – figure supplement 1A

Colorimetric

MRC5

Exposition 60sec

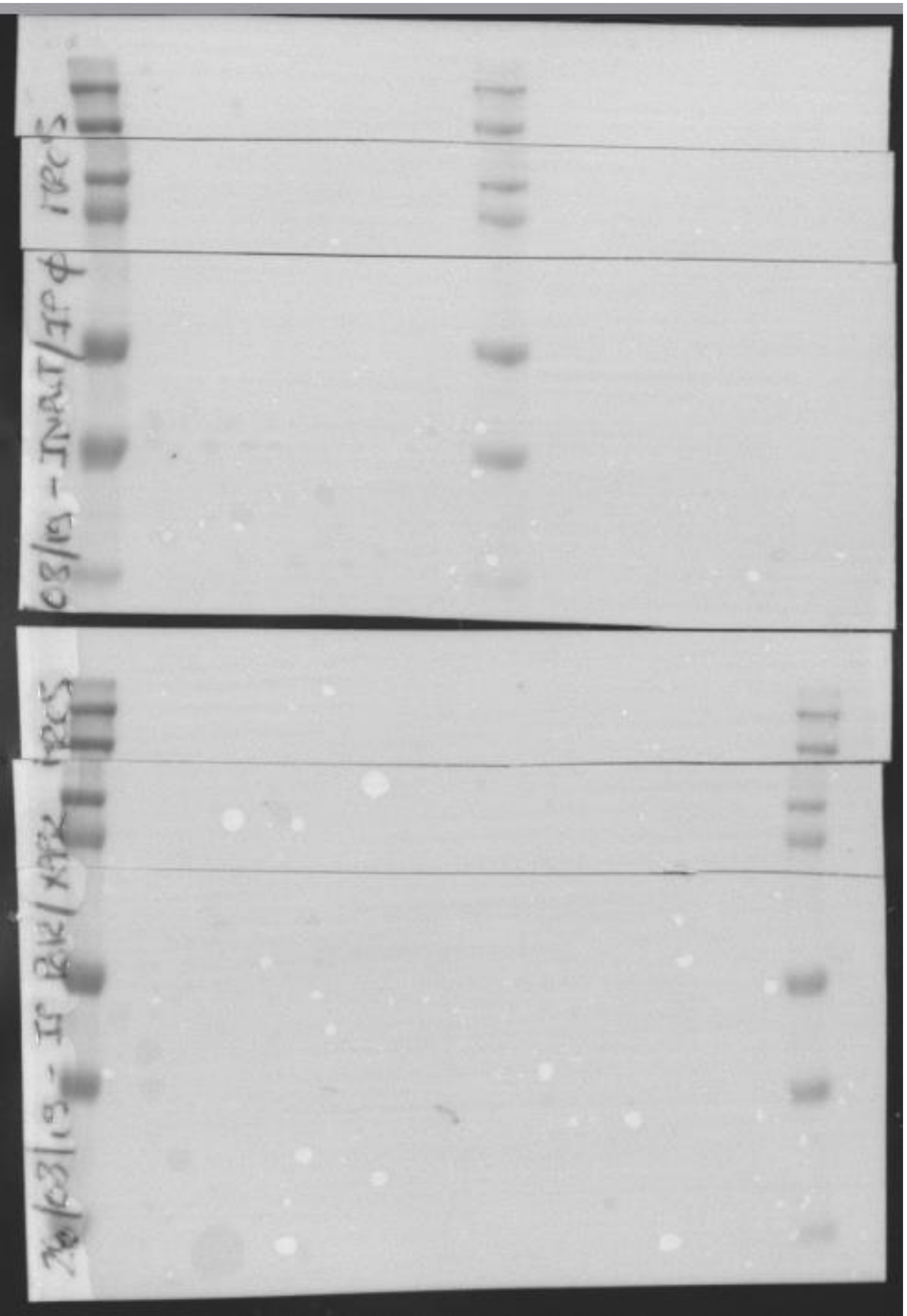

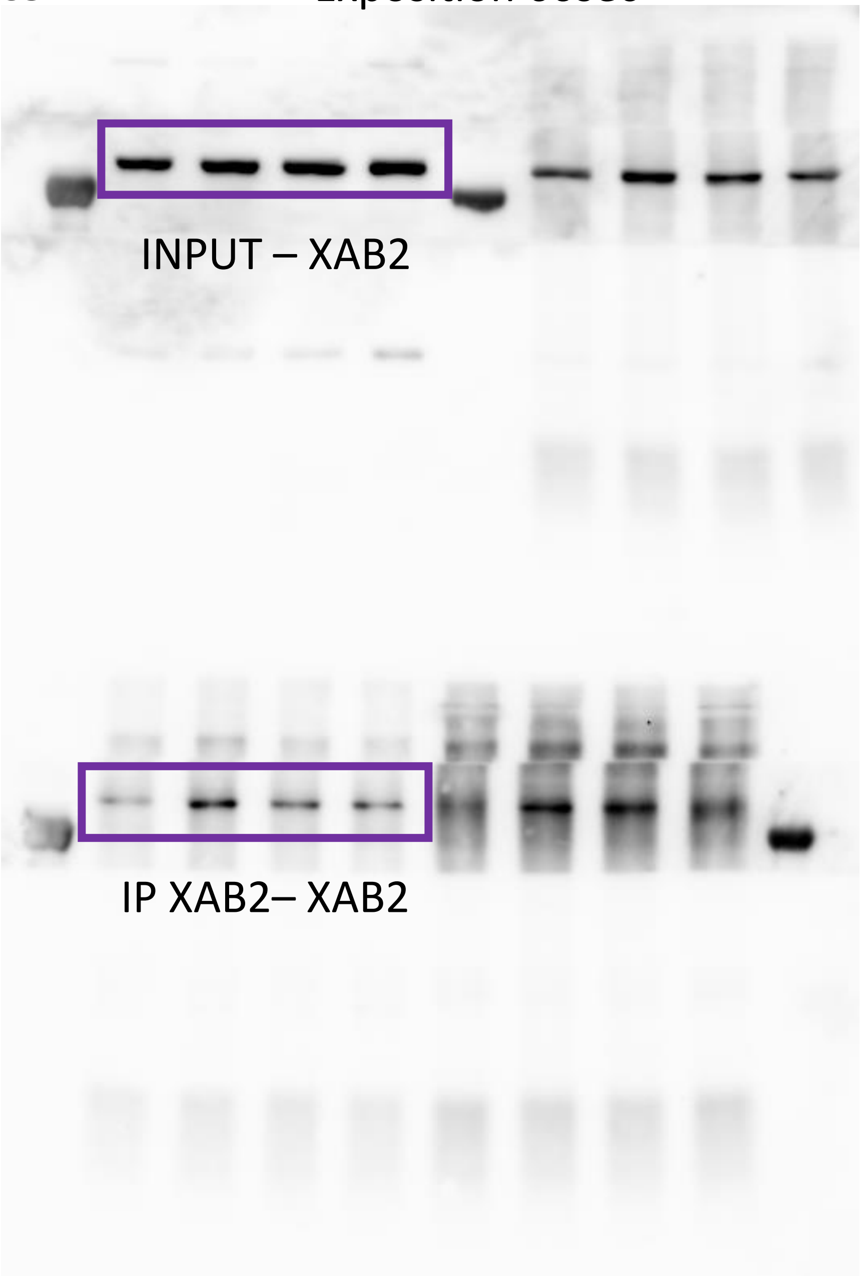

Figure 5 – figure supplement 1A

Colorimetric 2

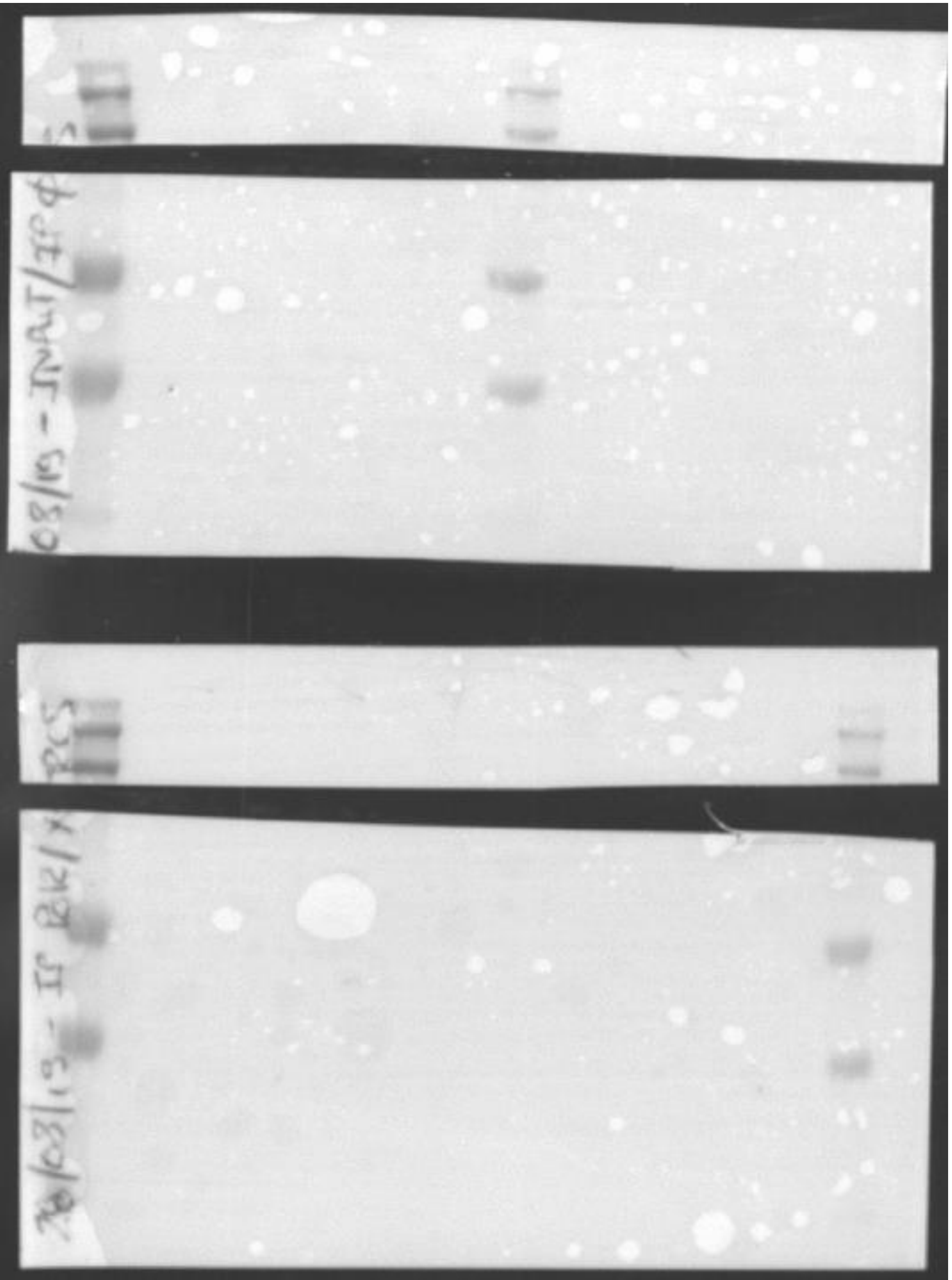

Exposition 1080sec -reblot

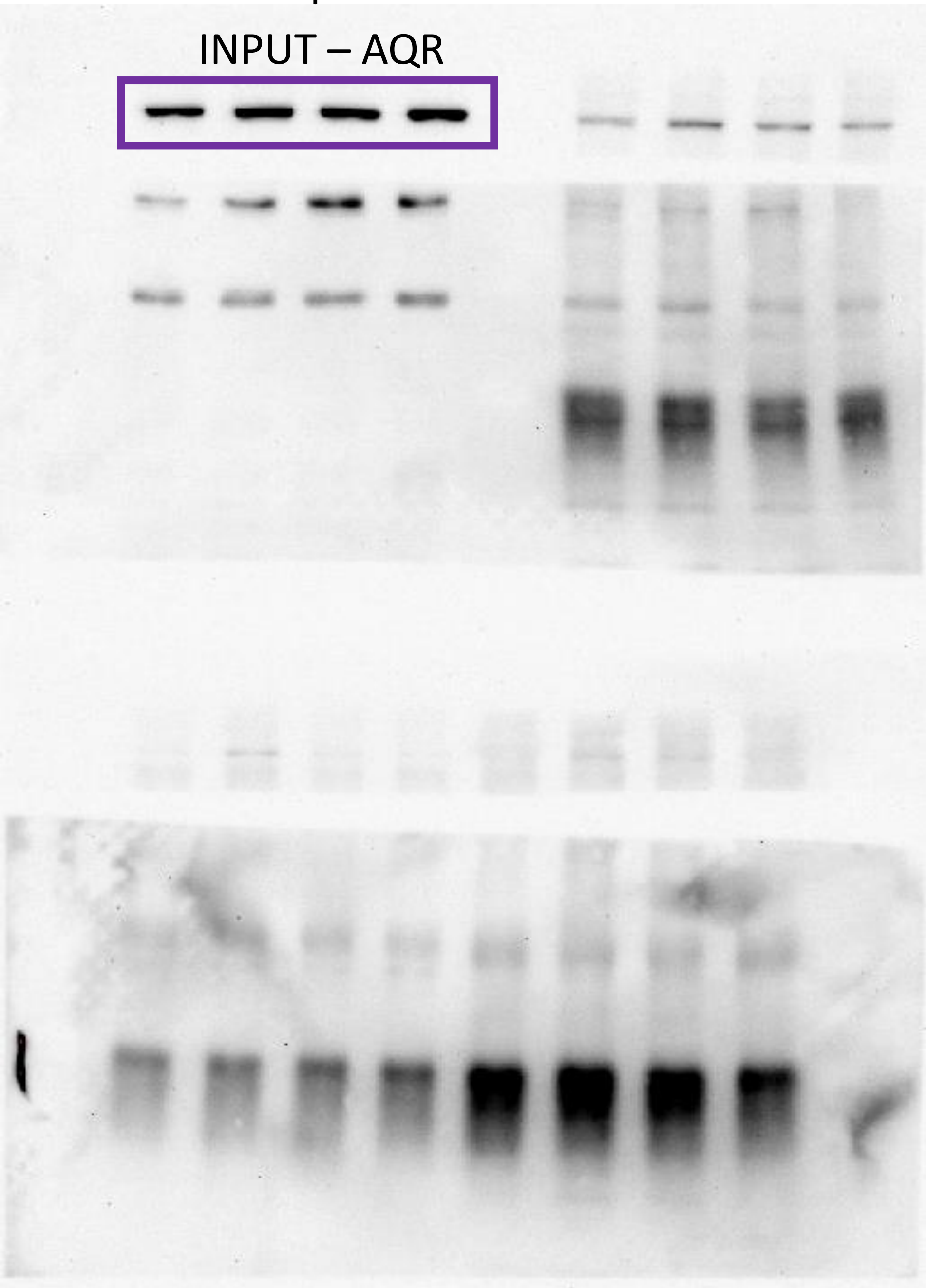

Figure 5 – figure supplement 1A

Colorimetric 3

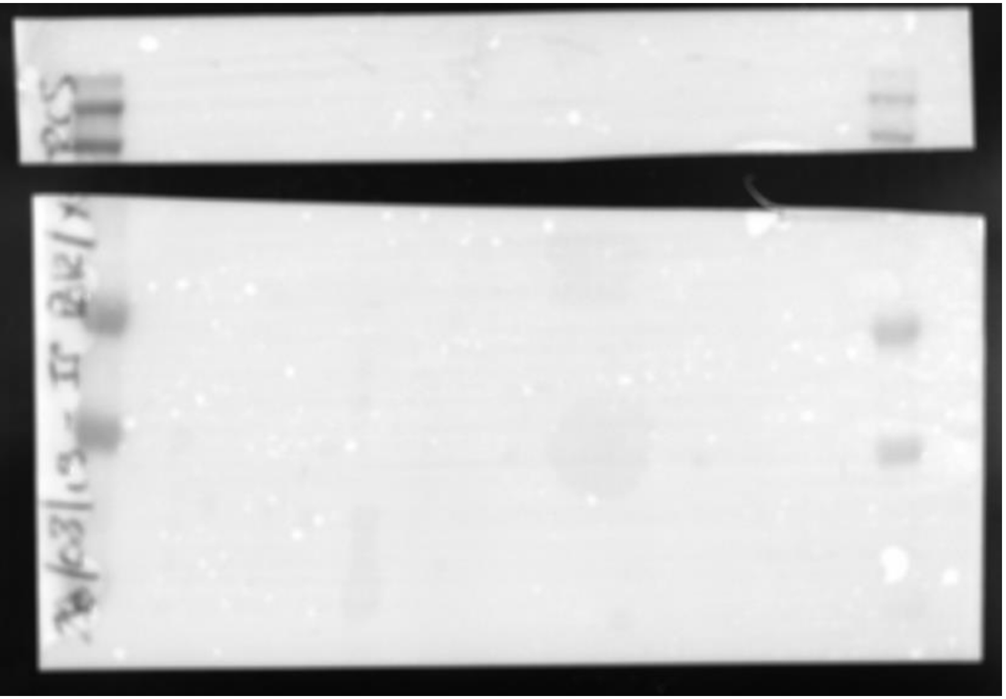

Exposition 1020sec – reblot - ECL++

IP XAB2 – AQR

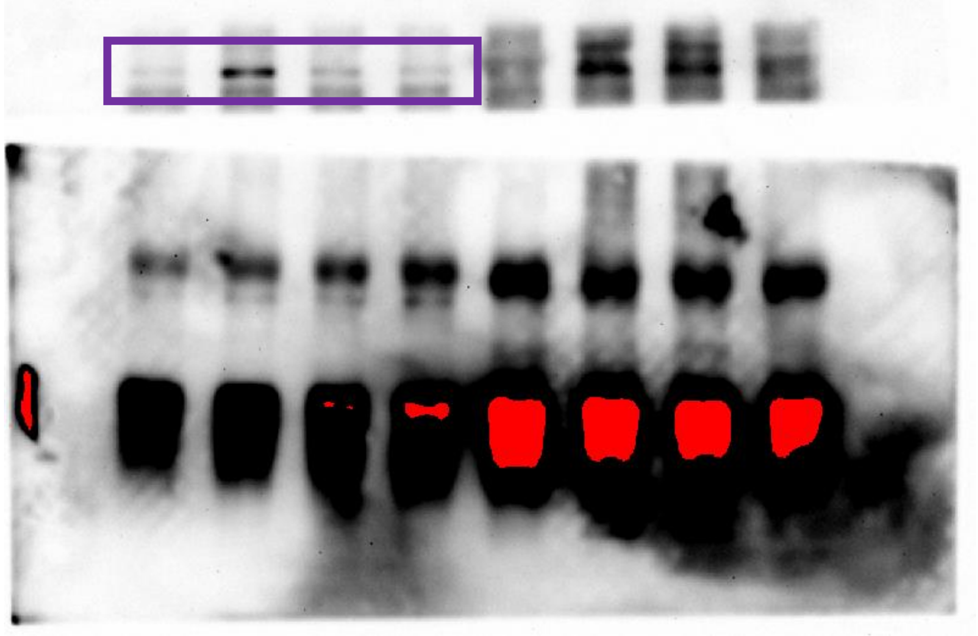

Supplement: Figure 5—figure supplement 1—source data 2. [file elife-77094-fig5-figsupp1-data2.pdf]
